# G-quadruplex in hepatitis B virus pregenomic RNA promotes its translation

**DOI:** 10.1016/j.jbc.2023.105151

**Published:** 2023-08-09

**Authors:** Jingjing Wang, Haiyan Huang, Kaitao Zhao, Yan Teng, Li Zhao, Zaichao Xu, Yingcheng Zheng, Lu Zhang, Conghui Li, Yurong Duan, Kaiwei Liang, Xiang Zhou, Xiaoming Cheng, Yuchen Xia

**Affiliations:** 1 State Key Laboratory of Virology and Hubei Province Key Laboratory of Allergy and Immunology, Institute of Medical Virology, TaiKang Center for Life and Medical Sciences, TaiKang Medical School, Wuhan University, Wuhan, China; 2 Key Laboratory of Biomedical Polymers-Ministry of Education, College of Chemistry and Molecular Sciences, Wuhan University, Wuhan, China; 3 Department of Pathophysiology, TaiKang Medical School, Wuhan University, Wuhan, China; 4 Department of Pathology, Hubei Clinical Center and Key Laboratory of Intestinal and Colorectal Diseases, Zhongnan Hospital of Wuhan University, Wuhan University, Wuhan, China; 5 Hubei Jiangxia Laboratory, Wuhan, China

**Keywords:** G-quadruplex, hepatitis B virus, pregenomic RNA, translation, PDS

## Abstract

Hepatitis B virus (HBV) is a hepatotropic DNA virus that has a very compact genome. Due to this genomic density, several distinct mechanisms are used to facilitate the viral life cycle. Recently, accumulating evidence show that G-quadruplex (G4) in different viruses play essential regulatory roles in key steps of the viral life cycle. Although G4 structures in the HBV genome have been reported, their function in HBV replication remains elusive. In this study, we treated an HBV replication-competent cell line and HBV-infected cells with the G4 structure stabilizer pyridostatin (PDS) and evaluated different HBV replication markers to better understand the role played by the G4. In both models, we found PDS had no effect on viral precore RNA (pcRNA) or pre-genomic RNA (pgRNA), but treatment did increase HBeAg/HBc ELISA reads and intracellular levels of viral core/capsid protein (HBc) in a dose-dependent manner, suggesting post-transcriptional regulation. To further dissect the mechanism of G4 involvement, we used *in vitro*-synthesized HBV pcRNA and pgRNA. Interestingly, we found PDS treatment only enhanced HBc expression from pgRNA but not HBeAg expression from pcRNA. Our bioinformatic analysis and CD spectroscopy revealed that pgRNA harbors a conserved G4 structure. Finally, we introduced point mutations in pgRNA to disrupt its G4 structure and observed the resulting mutant failed to respond to PDS treatment and decreased HBc level in *in vitro* translation assay. Taken together, our data demonstrate that HBV pgRNA contains a G4 structure that plays a vital role in the regulation of viral mRNA translation.

Although hepatitis B virus (HBV) prophylactic vaccine has been available for decades, more than 296 million people worldwide are currently living with chronic HBV infection (1). HBV is a hepatotropic DNA virus belonging to the Hepadnaviridae family. Its genome is a 3.2 kb, partially double-stranded and relaxed circular DNA (rcDNA), which contains four overlapping open reading frames (ORFs). After infection, the rcDNA is delivered into the nucleus of the cell and repaired to form covalently closed circular DNA (cccDNA), which serves as the template for viral transcription. Transcription yields four transcripts of different lengths of 3.5 kb precore RNA (pcRNA)/pre-genomic RNA (pgRNA), 2.4 kb preS1 RNA, 2.1 kb preS2/S RNA and 0.7 kb X RNA. The pcRNA is the template of HBeAg. HBeAg does not function during viral replication, while is a marker that reflects the level of cccDNA. HBV core (HBc) and polymerase are produced by pgRNA, HBc is a viral structure protein and assembled into nucleocapsids to promote the production of progeny viruses (2). HBV polymerase is DNA polymerase with reverse transcriptase activity that involves in viral genome replication processes. The preS1 and preS2/S translate viral surface protein-LHBs, MHBs and SHBs. In cytoplasm, HBc encapsidates pgRNA and polymerase to form the viral nucleocapsid in which pgRNA is reverse transcribed into negative-strand DNA, followed by the synthesis of incomplete positive strand to form viral rcDNA. The mature capsids contained rcDNA return to nucleus to expand cccDNA pool or enveloped to form progeny virions and released by multivesicular bodies (3, 4). Currently approved therapies for HBV infection, including nucleos(t)ide analogs and interferon α, are endowed with considerable adverse effects and limited effectiveness against the persistent cccDNA (5). In this regard, there is a pressing need to further dissect the HBV replication process for identification of novel therapeutic targets.

G-quadruplex (G4) refers to a four-stranded secondary structure formed by the interconnection of G-loops in the guanine-rich region through Hoogsteen-type hydrogen bonds pairing in DNA or RNA chains (6, 7). Previous studies have shown that DNA G4 is widely involved in a series of biological processes such as DNA replication (8), transcriptional regulation (9), and telomere maintenance (10). RNA G4 was reported to randomly distributed throughout the mRNA (5′UTR, ORF or 3′UTR), exerting different effects on translation (11, 12, 13, 14). To date, a number of small molecule ligands targeting G4 have been developed to study the function of G4, including 3,6,9-trisubstituted acridine compound (BRACO19) (15), 5,10,15,20-tetrakis-(N-methyl-4-pyridyl) porphine (TMPyP4) (16), pyridostatin (PDS) (17), and bisquinolinium derivatives (18). Among these, PDS is widely used because it exhibits high specificity to G4s and induces G4 stabilization (19).

In cancer research, certain G4 ligands, including Quarfloxin (CX-3543), CX-5461, and AS1411, have advanced to clinical trial stages (20, 21). Given advances in cancer, previous reports demonstrated that G4 is found in various viral genomes and mRNAs, and plays essential roles in viral transcription or translation regulation. For instance, in human immunodeficiency virus-1 (HIV-1), a large number of G4s are found not only on the viral genome but also on the integrated proviral genome, especially in the long terminal repeat (LTR) promoter (22, 23, 24) and *nef* coding region. Host factors such as nucleolin (25) and the human ribonucleoprotein A2/B1 (26) can bind with LTR G4 regions to weaken the promoter activity. In hepatitis C virus (HCV), the G4 in core gene is regulated by G4 ligands, and viral replication could be suppressed by G4 structure stabilizing (27). Furthermore, the herpes simplex virus (HSV) genome contains multiple clusters of repeated G4 (28, 29). Treatment of HSV-1 infected cells with BROCA-19 and TMPyP4 impairs virus production by inhibiting polymerase processing (28, 30). Additionally, the presence of G4 also affects the translation of some viral mRNAs. G4 in the nucleocapsid open reading frames of severe acute respiratory syndrome coronavirus 2 (SARS-CoV-2) has been reported and small molecule PDP (a PDS analogue) can impede nucleocapsid mRNA translation and reduce protein level by binding to G4 (31). Yet, study on G4 structure in HBV is still limited. Previous studies showed that G4s are presented in HBV precore promoter and genotype B preS2/S promoter (32, 33). While G4 upregulates preS2/S promoter activity and enhances the preS2/S gene transcription, G4 in precore promoter appears to only affect HBV antigen levels in the plasmid transfection model. However, the effect of G4 on the HBV infection is not fully illustrated.

In this study, we treated HepAD38 and HBV-infected HepG2-hNTCP-K7 cells with G4 structure stabilizer PDS. Subsequent analysis showed HBc protein expression, but not RNA level, was markedly enhanced by PDS. Further investigation by sequence alignment, analysis of bioinformatics, CD spectroscopy, transfection assay, and *in vitro* translation using *in vitro* transcribed (IVT) pgRNA confirmed that a conserved G4 on pgRNA regulates its translation efficiency.

## Results

### G4 stabilizing ligand PDS does not affect cccDNA transcription in HepAD38 cells

**Figure 1:**
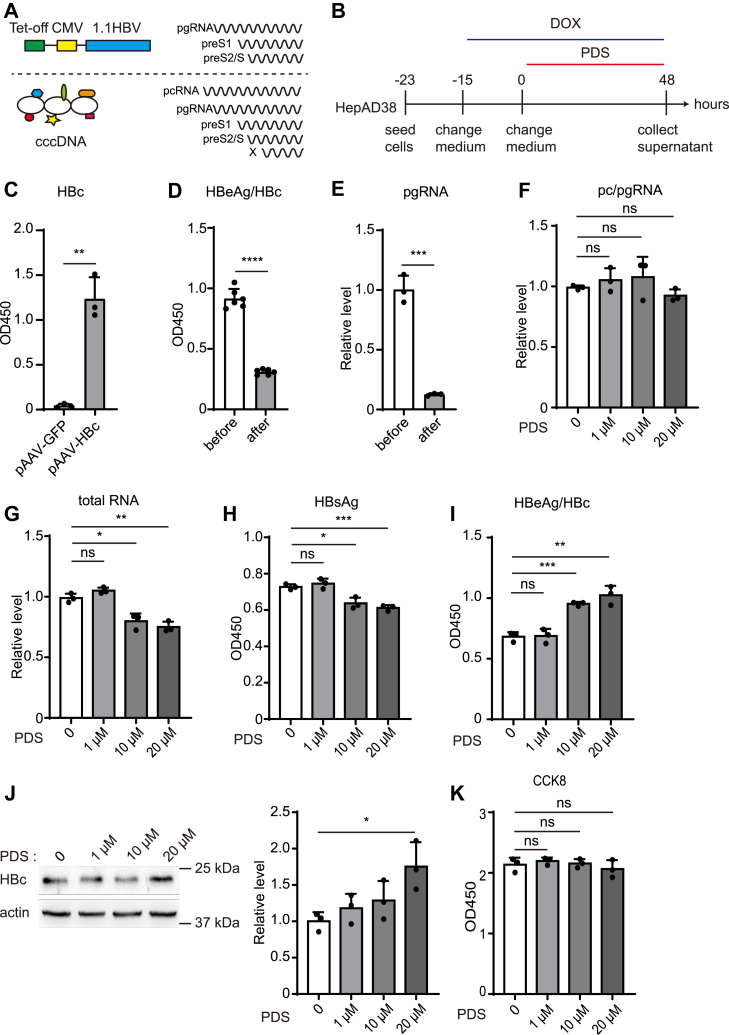
**The effect of PDS on the cccDNA transcription in HepAD38 cells.***A*, transcripts of the integrated HBV genome and cccDNA. *B*, experiment outline: HepAD38 was seeded in 24-well plates, and pretreated with doxycycline (DOX, 1 μg/ml) for 15 h. Afterwards, DOX and PDS were added for 48 h. *C*, pAAV-HBc was transfected into Huh7 cells, and HBc levels of cell lysates were determined by the commercial HBeAg ELISA kit. *D* and *E*, HBeAg/HBc in supernatant and intracellular pgRNA were measured by ELISA and RT-qPCR before and after pretreatment with DOX. *F* and *G*, HBV pc/pgRNA and total RNA were detected by RT-qPCR. *H* and *I*, HBsAg, and HBeAg/HBc were analyzed by ELISA after additional PDS treatment. *J*, the intracellular HBc was measured by Western blot and analyzed by Image J software. *K*, HepAD38 viability was detected 2 days after PDS treatment.

To examine the effect of G4 on HBV cccDNA transcription, HepAD38 cells containing an integrated 1.1-fold HBV genome under the control of tet-off regulatory element were used (34). HBV transcription proceeds from two distinct templates in this model, namely, cccDNA and the HBV genome integrated into the host chromosome (Fig. 1*A*). The integrated HBV genome transcribed pgRNA, preS1 and pres2/S where pgRNA production was controlled by regulatory elements (34). To examine whether PDS affected the transcription of cccDNA in HepAD38, doxycycline (DOX) was applied 15 h prior to co-treatment with PDS to shut down the pgRNA transcription from the integrated HBV genome, which meant that only cccDNA transcription remained (Fig. 1*B*). Since HBeAg and HBc process an overlapping ORF, the commercial HBeAg ELISA kit may detected an immunologic across interaction between them. To confirm this, ectopic expression of HBc in Huh7 cells could detected high levels of positive signals in cell lysates using HBeAg ELISA kit (Fig. 1*C*). The data indicated that HBc and HBeAg signal could not distinguished by the commercial kit. Therefore, we detected HBeAg/HBc in supernatant with the HBeAg ELISA kit. A significant decrease in HBeAg/HBc and pgRNA was observed after 15 h DOX treatment, indicating HBV transcription from genome was inactivated (Fig. 1, *D* and *E*). Henceforward, the cccDNA existing in the cells served as the only template for HBV transcription in the presence of DOX. After PDS treatment, there was no noticeable change detected in intracellular pc/pgRNA (Fig. 1*F*). And HBV total RNA and secreted HBsAg level exhibited a modest decrease (Fig. 1, *G* and *H*). Interestingly, HBeAg/HBc reads in supernatant increased in a dose-dependent manner (Fig. 1*I*). Similarly, intracellular HBc had a clear increase when treated with 20 μM PDS (Fig. 1*J*). In the meantime, cell viability was not affected (Fig. 1*K*). Taken together, PDS had no effect on the transcription of cccDNA but could affect viral protein translation.

### PDS influences HBV core protein in the infection system

**Figure 2:**
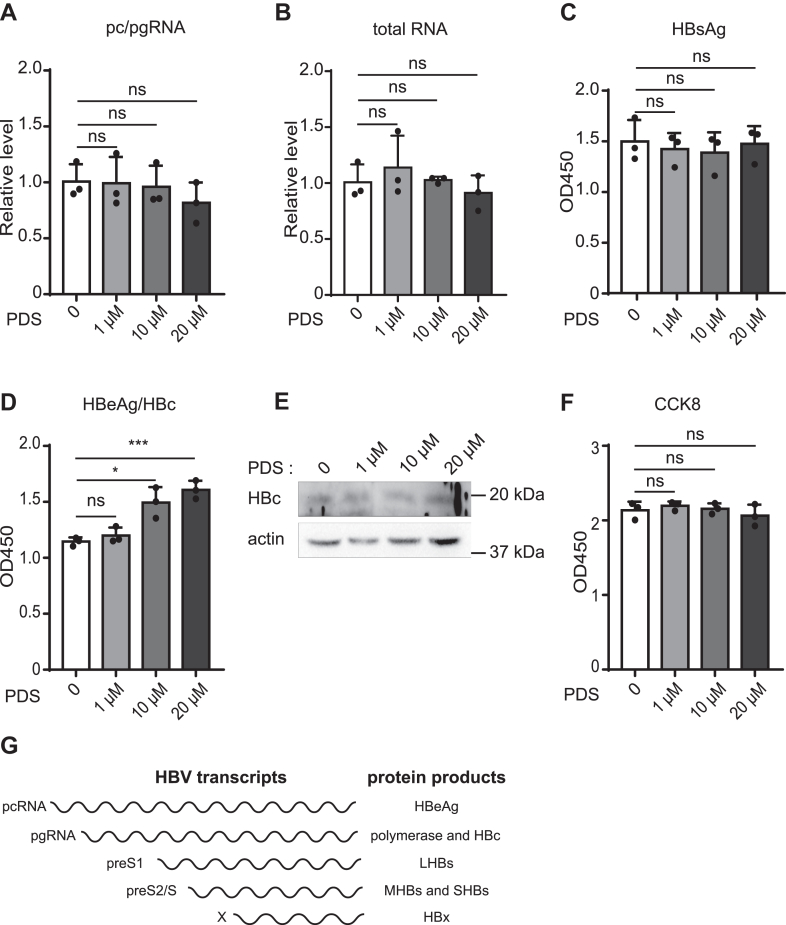
**The effect of PDS on cccDNA transcription in HBV-infected HepG2-hNTCP-K7 cells.** HepG2-hNTCP-K7 cells were infected with HBV at an MOI of 200, and then the cells were treated by PDS at 3 days post-infection (dpi). HBV infected HepG2-hNTCP-K7 with MOI 200, and PDS treated the cells at 3 days post-infection (dpi). We harvested supernatant and cells for the detection of HBV indicators in 7 dpi. *A* and *B*, at 7 dpi, cell total RNA was extracted according to the instruction. HBV total RNA (*A*) and pc/pgRNA (*B*) were quantitated by RT-qPCR. *C* and *D*, secreted HBsAg and HBeAg/HBc were measured by ELISA. *E*, changes in HBc level after treatment with different concentrations of PDS were analyzed by Western blot. The experiment was repeated three times. *F*, HepG2-hNTCP viability was determined 4 days after PDS treatment. *G*, HBV transcripts and the corresponding protein products.

As HepAD38 cells do not represent the viral full life cycle, we then used the HBV infection system to test the effect of PDS. HepG2-hNTCP-K7 cells were infected with HBV at a multiple of infection (MOI) of 200, and incubated with PDS starting from 3 days post-infection (dpi). At the transcriptional level, we observed no obvious changes in HBV RNAs by RT-qPCR (Fig. 2, *A* and *B*). While HBsAg showed no increase after PDS treatment, HBeAg/HBc levels were upregulated to a great extent (Fig. 2, *C* and *D*). Consistent with that, only minor upregulation of intracellular HBc was observed upon PDS treatment, which could be attributed to the fact that HBc may not have been readily detectable due to constraints on infection efficiency (Fig. 2*E*). The change in HBV markers was not caused by altered cell viability (Fig. 2*F*).

The disparities in the changes of HBV total RNA and HBsAg levels between the HepAD38 and infected systems could stem from variations in preS1/preS2/S arising from the integrated HBV genome. In the HBV life cycle, pcRNA is the translation template for HBeAg, while HBc is translated from pgRNA (Fig. 2*G*). Based on the results that the extracellular and intracellular HBV antigens were increased without affecting viral RNAs, we concluded that PDS had no effect on HBV cccDNA transcription but may affect pcRNA and/or pgRNA translation.

### PDS enhances pgRNA but not pcRNA translation

**Figure 3:**
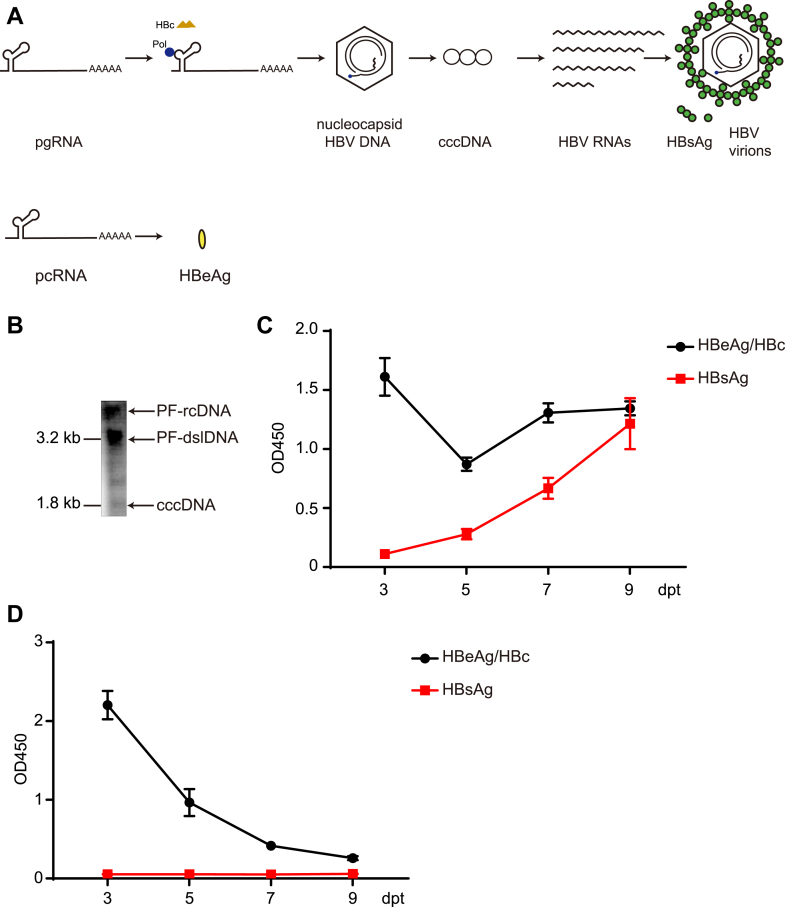
**Transfection *in vitro* transcription pgRNA and pcRNA in Huh7-hNTCP.***A*, the *in vitro* transcribed pgRNA was translated and reverse transcribed after transfection into cells and repaired to form cccDNA, which can complete the HBV life cycle. And pcRNA was translated into HBeAg. *B*, at 9 days post-transfection (dpt), hirt DNA was extracted to analyze HBV replication intermediates by Southern blot. *C* and *D*, HBeAg/HBc and HBsAg in supernatant were measured by ELISA from 3 dpt to 9 dpt in pgRNA and pcRNA transfection.

**Figure 4:**
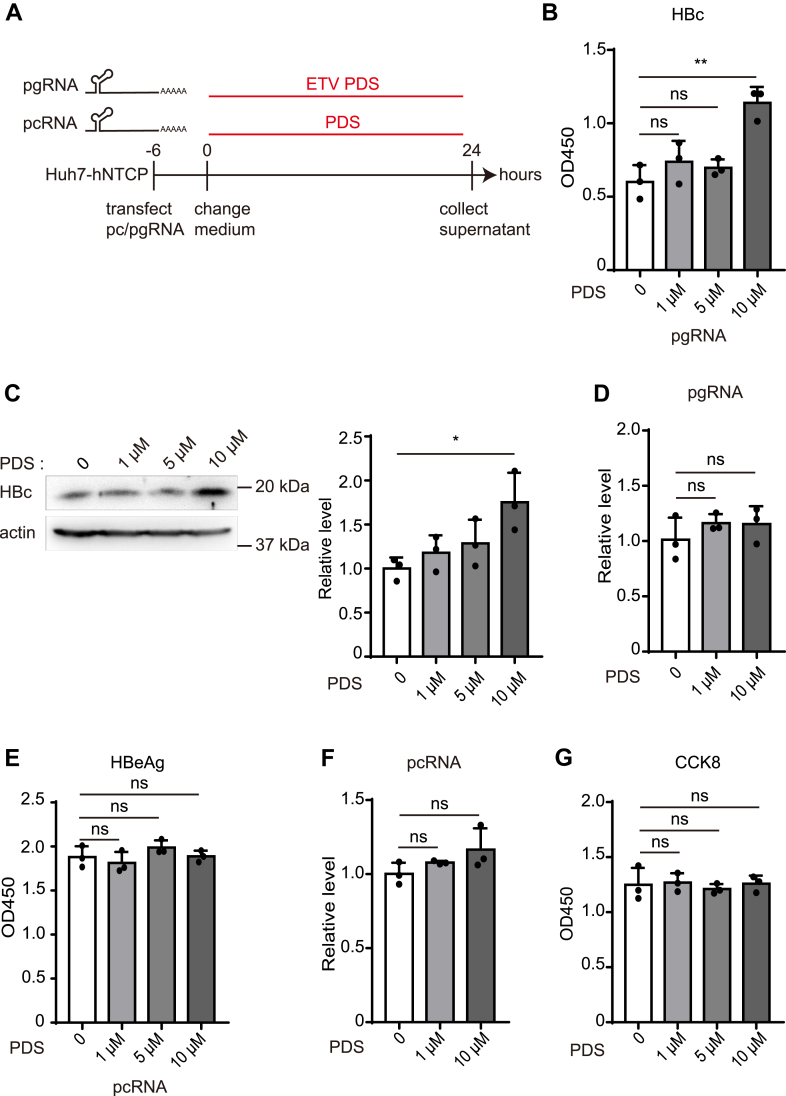
**The effect of PDS on pgRNA and pcRNA translation.***A*, after pgRNA and pcRNA transfection, Huh7-hNTCP were co-treated with ETV (0.5 μM) and PDS or only PDS for 24 h. *B*, *C* and *E*, HBc and HBeAg were detected by ELISA and Western blot after pgRNA or pcRNA transfection. Western blot was analyzed by Image J software. *D* and *F*, 24 h after PDS treatment, total RNA was extracted and we measured pgRNA and pcRNA by RT-qPCR. *G*, Huh7-hNTCP viability was analyzed at 24 h of PDS treatment.

As PDS is supposed to act through G4, we next analyzed the sequences of HBV RNAs. G4 analysis tool QGRS Mapper predicted there were potential G4 structures in both pgRNA and pcRNA (data not shown) (35). To evaluate the effects of G4 on translation efficiency of pgRNA and pcRNA, we obtained *in vitro* transcribed (IVT)-derived pgRNA and pcRNA, and transfected them individually into Huh7-hNTCP cells. As previously reported, the IVT pgRNA could be translated into HBc and polymerase, and then reverse transcribed into rcDNA, followed by cccDNA formation, full sets of viral gene transcription, and subsequent antigens and virions secretion (36, 37). On the other hand, the IVT pcRNA could only facilitate the production of HBeAg (Fig. 3*A*). Southern blot of HBV replication intermediates and ELISA of the HBeAg/HBc and HBsAg in supernatant confirmed that the transfected IVT pgRNA could resemble the replication of HBV (Fig. 3, *B* and *C*). The detection of HBeAg and HBsAg confirmed that the IVT pcRNA functioned as the translation template of HBeAg expression (Fig. 3*D*). To further validate the results, we also transfected the IVT pgRNA and pcRNA in Huh7-hNTCP cells and treated with ETV and PDS, or PDS only (Fig. 4*A*). ETV inhibits pgRNA reverse transcription, leaving the input IVT pgRNA as the only template for HBc production. Along the same line with the data obtained from HepAD38 and HBV infected HepG2-hNTCP-K7, ELISA and Western blot illustrated that HBc had about two-fold increase under 10 μM PDS treatment in pgRNA transfection group (Fig. 4, *B* and *C*). In addition, within the experimental timeframe, the unchanged levels of HBV RNA measured by RT-qPCR further supported that the increased HBc expression was caused by change in the translation efficiency under PDS treatment (Fig. 4*D*). In contrast, HBeAg in the supernatant had no significant change in pcRNA transfection group (Fig. 4*E*), although pcRNA itself was stable during treatment (Fig. 4*F*). Cell viability was also not affected by PDS for the duration of treatment (Fig. 4*G*).

### G4 existing in pgRNA promotes its translation efficiency

**Figure 5:**
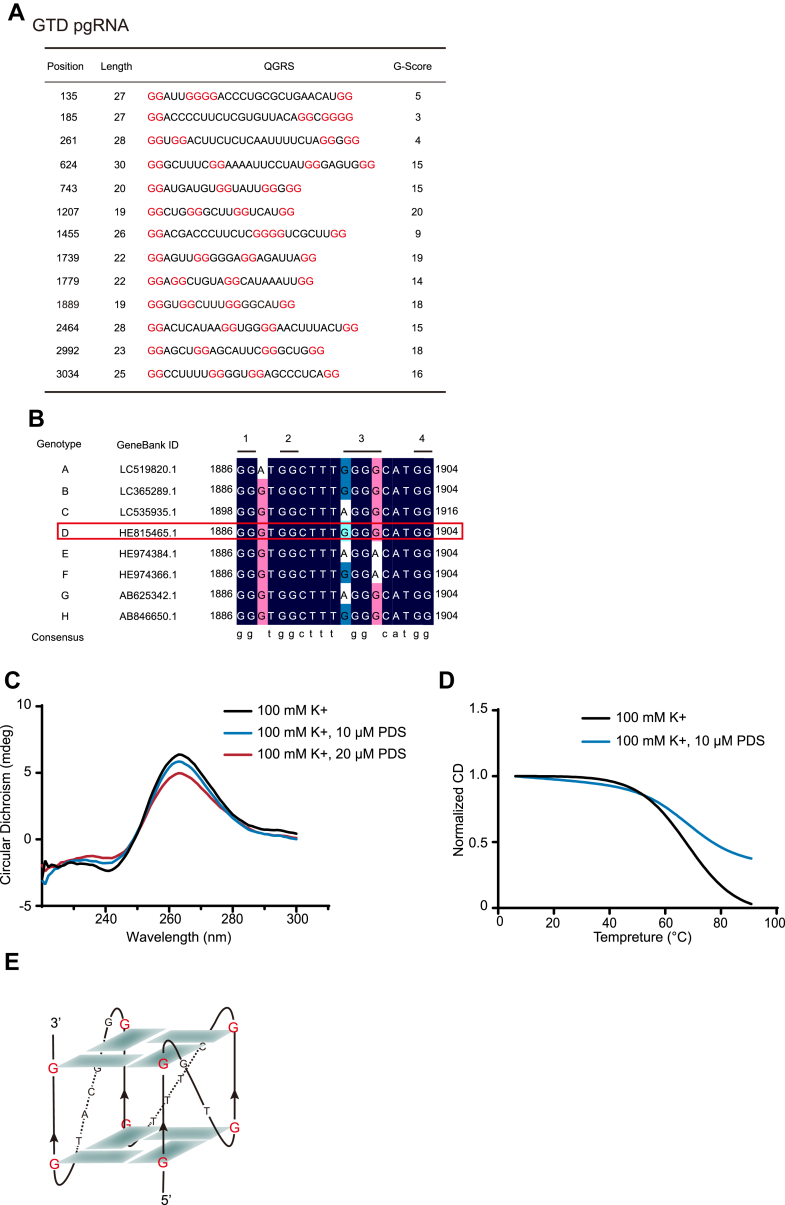
**pgRNA-G4 analysis, formation, and stabilization by PDS.***A*, the predicted possible G4 sites of genotype D pgRNA by QGRS Mapper. Red represents the G that participated in the formation of G4. *B*, conservative of G4 sequences across A-H genotype was analyzed by DNAMAN. *C*, CD spectra of pgRNA-G4 in the presence of PDS ranging from 0 to 20 μM. *D*, CD thermal melting curves of pgRNA-G4 (10 μM) in the absence or presence of PDS (10 μM). *E*, schematic representation of the proposed pgRNA-G4 structure.

It had been reported that G4 in 5′- or 3′-untranslated region (UTR) and ORF of mRNAs mainly repress cap-dependent translation, whereas G4 in 5′-UTR near internal ribosome entry site (IRES) likely enhances the IRES-mediated translation (38). To further verify the action mode of G4 on pgRNA translation, we analyzed the genotype D pgRNA used in the aforementioned experiments for possible G4 sites by QGRS Mapper. Notably, the predicted site “1889” locates at the epsilon loop which is near the 5′ UTR and across the ATG of HBc (Fig. 5*A*). Furthermore, this G4 sequence was conserved across different genotypes (Fig. 5*B*). Therefore, we focused on this site. To further confirm the existence of G4 structure in pgRNA, CD spectra of synthetic pgRNA-G4 was conducted. CD spectra indicated that pgRNA-G4 showed a positive peak around 264 nm and a negative peak close to 240 nm (Fig. 5*C*), which is the characteristic CD signature of a parallel G4 structure. In addition, PDS increased the thermal stability of pgRNA G4 structure, demonstrating the binding of PDS on pgRNA G4 structure (Fig. 5*D*). After interacting with PDS, positive and negative CD peaks only decreased slightly in intensity without position change, implying that PDS treatment did not alter parallel structure of pgRNA G4 (Fig. 5*C*). The decrease of the positive and negative peak was due to the partial unwind of G4s, this may cause by the interaction between PDS and pgRNA-G4 as previous research reported similar phenomenon in SARS-CoV-2 RNA G4 (39). Based on the abovementioned results, we proposed that pgRNA-G4 forms a stable parallel G4 structure as shown in Figure 5*E*.

**Figure 6:**
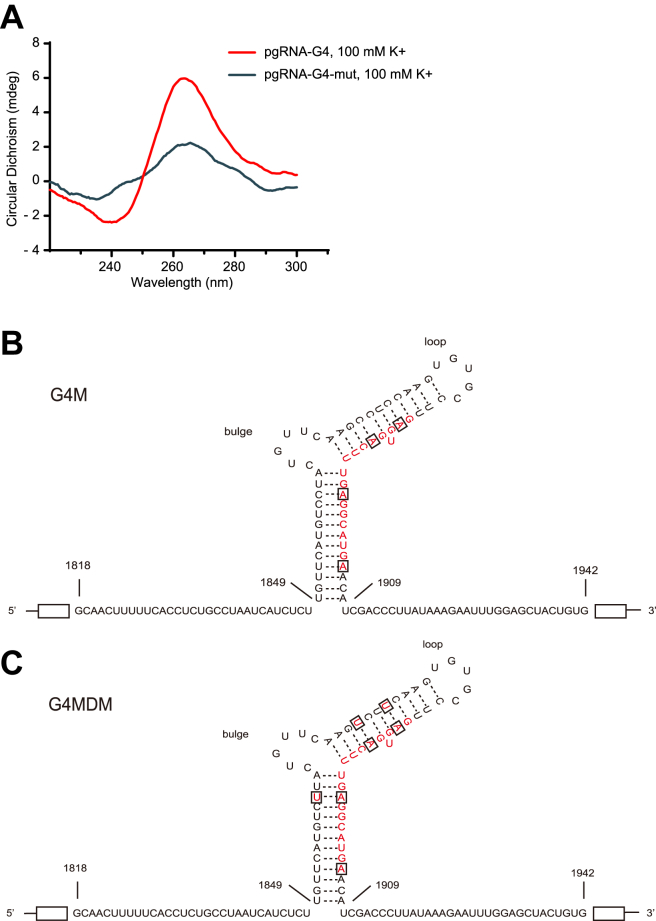
**Site mutations on pgRNA.***A*, CD spectra of pgRNA-G4 WT and mutant. *B*, G4M pgRNA that G was replaced by A. *C*, G4MDM pgRNA which G was mutated to A as shown, and the paired C was mutated to U.

**Figure 7:**
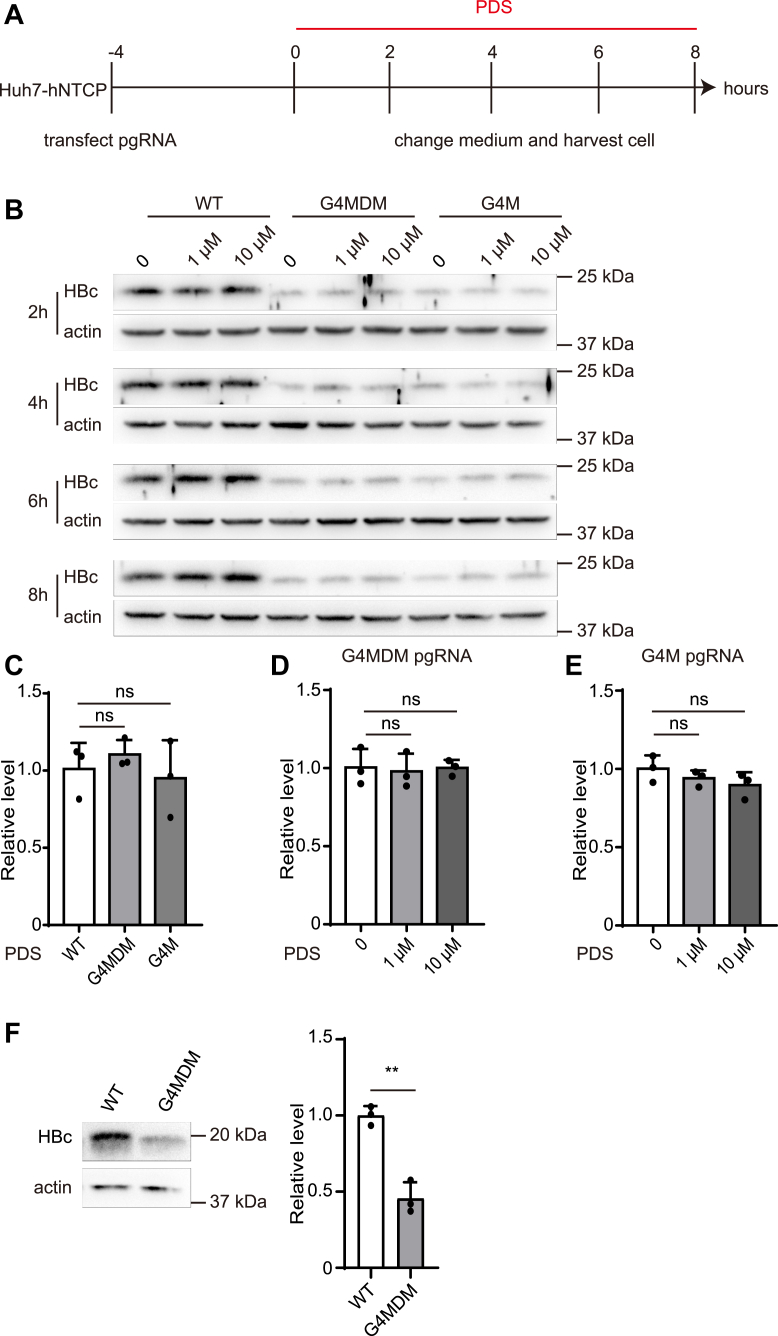
**PDS and G4 mutation on pgRNA translation at different time points after transfection.***A*, 4 h after IVT pgRNA transfection in Huh7-hNTCP, the medium was changed and PDS was added, then the cells were harvested every 2 h. *B*, changes in intracellular HBc levels every 2 h were detected by Western blot. *C*, *D*, and *E*, 6 h after IVT pgRNA transfection and PDS treatment, cellular total RNA was extracted and analyzed pgRNA levels by RT-qPCR. *F*, *in vitro* translation HBc levels were measured by Western blot. Grayscales were analyzed by Image J software.

To conclude the role of G4 in pgRNA translation, we introduced mutations to disrupt the G4 structure. The pgRNA-G4-mut is a mutation of the predicted 19 bp sequence-pgRNA-G4 carried G to A mutations at positions 2, 6, 12, and 19. CD spectra showed that pgRNA-G4-mut had lower positive and negative peaks compared with wide-type (WT) pgRNA-G4 (Fig. 6*A*). The pgRNA that coincided with the location of the pgRNA-G4-mut was named G4M (Fig. 6*B*). The other mutant was named as G4MDM, containing the same G to A mutations and additional C to U mutations in the complementing strand to maintain the 2D structure of epsilon loop (Fig. 6*C*). We then transfected WT or mutant pgRNA into Huh7-hNTCP cells, and closely monitored HBc levels every 2 h after PDS treatment (Fig. 7*A*). As shown in Figure 7*B*, compared with WT, mutations in the G4 region markedly attenuated HBc expression. As expected, WT pgRNA responded to PDS treatment as demonstrated by the increased expression of HBc at 6 h post treatment (Fig. 7*B*). While a slight change in the HBc level was observed with G4M mutant, G4MDM pgRNA mutant failed to respond to PDS treatment (Fig. 7*B*). Moreover, neither mutagenesis nor PDS treatment altered pgRNA stability (Fig. 7, *C*–*E*). In addition, we utilized the rabbit reticulocyte lysate system to use pgRNA as template for *in vitro* translation. Western blot verified that disrupting the G4 structure affected the HBc level (Fig. 7*F*). Together, these results indicated that G4 in pgRNA enhanced its translation efficiency.

## Discussion

In this study, we reported the presence of a conserved G4 motif in the pgRNA and pcRNA, demonstrated a regulatory role for this secondary structure in pgRNA translation but not pcRNA, and proved that the G4 within the pgRNA positively regulated HBc translation in HepAD38 cell line, HBV infection system and *in vitro*-synthesized RNA transfection system. CD spectra and melting experiment demonstrated pgRNA-G4 structure and the interaction with PDS. To verify the significance of G4 in pgRNA, we introduced mutations to disrupt the G4 motif which subsequently showed to impair pgRNA translation. Additionally, an increase in the translation efficiency after stabilization of the G4 motif with ligand PDS and a decrease of HBc in *in vitro* translation assay supported our finding that G4 formation was beneficial for pgRNA translation. Furthermore, mutations and PDS treatment did not affect pgRNA stability.

Previous studies have shown that G4 formation in RNA is implicated in several biological processes, including transcription termination and polyadenylation (40, 41), pre-mRNA splicing (42, 43), and translation (11, 12, 44, 45). During transcription, G4 sequences serve as terminator signals by inducing RNA Pol II transcriptional pausing. The mechanism may involve the formation and resolution of the G-quartet (40). G4 structures are discovered in splicing sites of a list of genes, and they function as regulatory elements of splicing reaction. G-rich splicing regulatory sequences are associated with specific splicing regulator (SR) proteins to enhance the splicing process (46). The fragile X mental retardation protein (FMRP) enhanced *FMR1* mRNA splicing through interaction with G4 (47). Translation is one of the most fundamental processes in RNA biology. And G4 located at 5′UTR, ORF or 3′UTR are known to have different effects on translation. For most eukaryotic mRNAs, translation initiation is dependent on the 7-methylguanosine cap in 5′UTR. However, there is a cap-independent mode of translation driven by IRES for some viral and host mRNAs. Several studies showed that G4s in 5′UTR correlated with translation repression of various mRNAs including *TRF2* (45) and *NARS* (11). And the possible reason that G4 in 5′UTR decreased protein expression in cap-dependent translation was by inhibiting ribosome scanning processes. For cap-independent translation initiated by IRES, G4 promoted translation efficiency, such as *FGF2* and *VEGF* mRNA (12, 38), but the involved mechanism was unclear. The G4 in CDS affects other processes in translation. Mura *et al.* revealed that the Epstein–Barr virus-encoded nuclear antigen 1 (EBNA1) mRNA CDS formed a G4 structure, and antisense oligonucleotides destabilized G4 formation. They demonstrated this structure impeded translation elongation (13). Moreover, 3′UTR is an essential regulatory element for mRNA stability and translation. Previous study reported a conserved G4 located in the 3′UTR of *PIM1* inhibited translation (48). The chosen G4 stabilizer PDS was reported to target host telomeres, hence largely excluding the possibility that PDS affected virus replication by altering gene expression of some host factors (17). In addition, PDS has rotatable bonds allowing it to bind different forms of G4 and has been used to study the G4 structure on a variety of viruses, including EBV (13), ZIKV (49), and HBV (32). In this study, G4 in pgRNA is located at the epsilon loop that may serve as IRES. IRES-mediated translation is cap independent and G4 located in the IRES region will promotes protein expression. This may allow virus to compete with the host for survival resources and achieve symbiosis with cells after reaching equilibrium.

In recent years, Biswas *et al.* (32) demonstrated that a G4 motif in an envelope gene promoter regulates transcription and virion secretion in genotype B HBV. *Meier-Stephenson* team identified a conserved G4 structure in the precore promoter region of HBV cccDNA using the HBV plasmid transfection system (33). Afterall, the role of G4 in the full HBV life cycle remains elusive. By using HBV stable cell line, infection system, and IVT-transfection models, we demonstrated that the G4 in HBV pgRNA enhanced its translation efficiency but had no effect on pcRNA. Agents targeting G4 may positively or negatively regulate HBV viral proteins. It has been demonstrated that a series of disease-related genes are regulated by G4 (11, 43, 50), and telomeric G4 stabilizers are considered as novel drugs for cancer treatment (21). For HBV-related liver cancer, this kind of drugs should be carefully evaluated considering their potential effect on HBV. In summary, we have uncovered a previously unknown mechanism governing the HBV life cycle and provided evidence that G4 ligand anticancer drugs could elicit HBV protein expression.

## Experimental procedures

### Cell culture

Huh7-hNTCP and Huh7 were gifted by Prof. Xinwen Chen from the Wuhan Institute of Virology, Chinese Academy of Science. HepAD38 and HepG2-hNTCP cells were gifted by Prof. Ulrike Protzer from Technical University of Munich, Germany. Cells were maintained in the Dulbecco’s modified Eagle’s medium (DMEM) (Gibco, SH30022.01) supplemented with 10% fetal bovine serum (FBS) (Lonsera, Uruguay, S711-001S) and 100 U/ml penicillin/streptomycin (Gibco, 15070063), and cultured at 37 °C in a 5% CO_2_ incubator.

### HBV infection

HBV production and infection process were performed as described previously (51). HBV stable replication cell line HepAD38 was used to produce HBV virions. The medium containing HBV was first filtered through a 0.22 μm filter (Millipore, SLGPR33RB) and then concentrated with 100 kDa Ultra Centrifugal Filters (Millipore, UFC710008). For HBV infection, HepG2-hNTCP-K7 cells were seeded, pre-treated with culture medium containing 2.5% DMSO (Sigma-Aldrich, D2650) for 2 days prior to inoculation with about 200 HBV genome equivalents per cell (MOI 200) in the presence of 4% PEG8000 (Sigma-Aldrich, 89510). After 24 h, the infection inoculum was removed, and cells were washed three times with PBS (Gibco, C10010500BT), followed by routine maintenance.

### Plasmids

The pcDNA3.1-pgRNA and pcDNA3.1-pcRNA plasmids were constructed. Site mutations were introduced into predicted sites on pcDNA3.1-pgRNA to disrupt the G4 structure. The used primers of pcDNA3.1 pcRNA and pgRNA mutant plasmids are as follows. pcRNA fwd1: GCAGTACATCAAGTGTATCATATGCCAAGTACGCCCCCTATT; pcRNA rev1: GGTGCGCAGACCAATTTATGCCTACCTCCCTATAGTGAGT; pgRNA fwd2: ATTGGTCTGCGCACCAGCACCATGCAACTTTTTCACCTCT; pgRNA rev2: GTTTGGTGGAAGGTTGTGGAATTCCACTGCATGGCCTGAGGAT. G4M fwd: TGCCTTGAGTGACTTTGAGGCATGAACATC; G4M rev: GATGTTCATGCCTCAAAGTCACTCAAGGCA. The pcDNA3.1 pgRNA G4MDM used pcDNA3.1 pgRNA G4M as a template. G4MDM fwd: TCATGTCTTACTGTTCAAGTCTTCAAGCTG; G4MDM rev: GATGTTCATGCCTCAAAGTCACTCAAGGCA. pAAV-GFP and pAAV-HBc were preserved by the laboratory.

### *In vitro* transcription HBV pgRNA and pcRNA

*In vitro* transcription of HBV pgRNA and pcRNA was performed according to a previous study (37). Briefly, 2 μg linearized plasmids of pcDNA3.1-pgRNA (WT, G4MDM, and G4M) were used to produce pgRNA using the T7 mScript Standard mRNA Production System (CELLSCRIPT, C-MSC100625) according to the manufacturer’s instructions.

### IVT-RNA transfection

Huh7-hNTCP cells were seeded in 24-well plates, and transfected when reached about 90 to 100% confluence. The cells were washed two times with PBS and one time with Opti-MEM. Afterwards, 250 μl Opti-MEM was added. Transfection reagent Lipofectamine 2000 (Invitrogen, USA, 11668019) and RNA were mixed in a ratio of 5:1 (μl: μg) in Opti-MEM and incubated at room temperature for 20 min. The mixture was then added to the medium and spinoculated by centrifugation at 1000*g* for 30 min at 37 °C. The medium was removed after 2 to 6 h and replaced with fresh culture medium contained PDS (TOPSCIENCE, China, T4457).

### Cell viability assay

Cell viability was measured by Cell Counting Kit-8 (Biosharp, BS305) according to the manufacturer’s protocol. Briefly, cells were seeded in 96-well plates, and treated with PDS with corresponding time. Following the addition of CCK-8 reagent at 1:10 dilution in culture medium, cells were further incubated for 0.5 to 2 h in the incubator. We finally measured the absorbance at 450 nm with the microplate reader (HIDEX, 425-301).

### Western blot

Cell extracts were prepared by lysis buffer (50 mM Tris-HCl pH 8.0, 1 mM EGTA, 1 mM EDTA, 150 mM NaCl, 1% Triton-X-100, and proteinase inhibitor) at 4 °C and shook for 30 min. And the cell lysates were centrifuged at 10,000*g* for 10 min at 4 °C. The protein concentration was analyzed by a Bradford Assay Kit (Bio-Rad, 5000205). 30 μg cell lysates were used to perform the SDS-PAGE gel electrophoresis and subsequently transferred to a PVDF membrane (Millipore, SLGV013SL). PVDF membranes were blocked with 5% skim milk in Tris buffered saline with 0.1% Tween 20 (TBST) before antibody incubation. The following primary antibodies were used: anti-HBc (laboratory preservation), anti-β-actin (Cell Signaling Technology, 4970). Anti-rabbit IgG (HRP-linked Antibody) (Cell Signaling technology, 7074) was used as the secondary antibody.

### RNA extraction and quantitative PCR analysis

Total cellular RNA was isolated with RNApure Tissue & Cell Kit (COWIN Biosciences, China, CW0581) according to the manufacturer’s instructions. 500 ng of total RNA was reverse transcribed into cDNA using ReverTra Ace qPCR RT Master Mix (TOYOBO BIOTECH CO, FSQ-301) and then was measured by real-time PCR with FastStart Essential DNA Green Master (Roche, Germany, 6924204001). The following primers were used: for HBV total RNA: sense primer, 5′-CCGTCTGTGCCTTCTCATCTGC-3′, anti-sense primer, 5′-ACCAATTTATGCCTACAGCCTCC-3′; for HBV pgRNA/pcRNA: sense primer, 5′-CTGGGTGGGTGTTAATTTGG-3′, anti-sense primer, 5′-TAAGCTGGAGGAGTGCGAAT-3′; for human *ACTB*: sense primer, 5′-ATCGTGCGTGACATTAAGGAG, antisense primer, 5′-GGAAGGAAGGCTGGAAGAGT. The data were normalized to level of *ACTB* mRNA in each individual sample. 2−ΔΔCt method was used to calculate relative expression changes.

### Enzyme-linked immunosorbent assay

The levels of HBsAg and HBeAg in the culture medium were measured by ELISA (Shanghai Kehua Bio-Engineering Co) according to the manufacturer’s instructions.

### HBV hirt DNA extraction and detection by Southern blot

HBV hirt DNA extraction and detection were performed as described previously (52, 53). Briefly, cells from one well of six-well plates were lysed in 1.5 ml TE buffer (10:10) (10 mM Tris-HCl, pH 7.5, and 10 mM EDTA) supplemented with 100 μl 10% SDS for 30 min at room temperature, then the lysate was collected. Following the precipitation of proteins, protein-associated DNA was added with 400 μl 5 M NaCl at a final concentration of 1 M for at least 16 h at 4 °C. The protein precipitation was removed by centrifugation. DNA was extracted by multiple phenol and phenol/chloroform extractions and finally dissolved in TE buffer (10:1) (10 mM Tris-HCl, pH 7.5 and 1 mM EDTA). The DNA sample was subjected to 1.2% (wt/vol) agarose gel to perform the gel electrophoresis at 25 V overnight. The gel was subsequently treated with depurination buffer (0.2 M HCl) for 10 min, denaturing buffer (0.5 M NaOH, 1.5 M NaCl) for 1 h and neutralization buffer (1.5 M NaCl, 1 M Tris–HCl, pH 7.4) for 1 h in turn. After transferring the DNA products to nylon membrane (GE Healthcare Life sciences, RPN303B) overnight, HBV cccDNA was detected by hybridization with a ^32^P-labeled HBV DNA probe. Hybridization signals were analyzed by Typhoon FLA 9500 imager (GE Healthcare Life sciences).

### CD spectroscopy

CD spectra and CD melting experiments were performed on a CD spectrophotometer (Applied Photophysics, UK) equipped with a temperature-controlled water—bath using a quartz cuvette having a 1 mm optical path length and a sample volume of 200 μl. RNA oligos were annealed before measuring the spectra by heating the sample to 95 °C for 5 min in the buffer (10 mM Tris–HCl, 100 mM KCl and pH 7.0) then slowly cooled to room temperature. Annealed RNA and PDS (0, 10 μM and 20 μM) were incubated together at 4 °C for 24 h. The CD spectra was recorded for the wavelength range of 200 nm to 340 nm with a step size of 0.5 nm, a time-per-point of 0.5 s and a bandwidth of 2 nm. For CD melting experiment, the data was collected at 264 nm for a temperature range of 4 °C to 95 °C with a heating rate of 1 °C/min and a time-per-point of 5 s. RNAs were synthesized by Shanghai Sangon Biological Engineering Technology & Services. The pgRNA-G4-WT is GGGUGGCUUUGGGGCAUGG, and pgRNA-G4-mutant is GAGUGACUUUGAGGCAUGA.

### *In vitro* translation

WT and mutation pgRNA *in vitro* translation were performed with Flexi Rabbit Reticulocyte Lysate System (Promega Corporation, L4540) according to the manufacturer’s protocol. Lysates were then subjected to Western blot.

### Statistical analyses

Each point in all graphs represented a biological replicate of one experiment. Statistical significance was analyzed by *t* test using GraphPad Prism 7. ∗∗∗*p* < 0.001; ∗∗*p* < 0.01; ∗*p* < 0.05; ns, not significant. Dose-dependent results were analyzed by one-way ANOVA.

## Data availability

The datasets generated during and/or analyzed during the current study are available from the corresponding author upon reasonable request.

## Conflict of interest

The authors declare that they have no conflicts of interest with the contents of this article.
